# Multidimensional burden of low back pain: A prospective cross sectional study of patient-reported outcomes and sociodemographic factors at a tertiary neurosurgical center

**DOI:** 10.1016/j.bas.2025.105905

**Published:** 2025-12-13

**Authors:** Pavlina Lenga, Robin Fleige, Max Christian Blumenstock, Matthias Ganzinger, Sebastian Ille, Martin Dugas, Sandro M. Krieg

**Affiliations:** a Department of Neurosurgery, Heidelberg University Hospital, Heidelberg, Germany; b Institute of Medical Informatics, Heidelberg University Hospital, Heidelberg, Germany

**Keywords:** Low back pain, PROMs, Depression, Degenerative lumbar disease

## Abstract

**Introduction:**

Low back pain (LBP) significantly impacts patients’ physical function, mental health, ***erectile dysfunction (ED) in males***, and occupational status. However, comprehensive prospective assessments of these interrelated dimensions in specialized neurosurgical outpatient settings remain sparse.

Research Question: This study aimed to prospectively evaluate functional disability, depression, erectile dysfunction (ED), occupational impairment, and their associations with demographic and lifestyle factors among patients presenting to a neurosurgical outpatient clinic.

**Materials and methods:**

Between February and June 2025, we prospectively enrolled 247 consecutive patients (mean age 60.4 ± 16.0 years, 53.8 % male) presenting to the neurosurgical outpatient clinic at Heidelberg University Hospital with degenerative lumbar spine disorders, of whom 110 (44.5 %) subsequently underwent decompression surgery. Patients completed validated patient-reported outcome measures (PROMs), including pain intensity (Numeric Rating Scale [NRS]), functional disability (Oswestry Disability Index [ODI]), depression (PHQ-9), and erectile dysfunction (IIEF-5). Correlation analyses and regression models assessed associations among these outcomes and relevant demographic and lifestyle variables.

**Results:**

Patients reported high pain intensity (NRS 7.0 ± 2.2), severe functional disability (ODI 57.4 ± 16.5; 82.2 % severely disabled or worse), frequent moderate-to-severe depressive symptoms (42.2 %), and prevalent erectile dysfunction among males (55 %). Multivariate analyses identified depression severity (PHQ-9; B = 1.30, p < 0.001), older age (B = 0.20, p = 0.045), and surgery (B = 6.13, p = 0.049) as significant predictors of greater disability. Erectile dysfunction severity in males was independently associated with higher disability (B = −0.18, p = 0.002) and older age (B = −0.32, p < 0.001). Undergoing surgery was significantly predicted by higher baseline disability (ODI; OR = 1.04, p = 0.032) and private insurance (OR = 22.39, p < 0.001). Among working-age patients (≤65 years, n = 142), occupational disability was notably high, with 39.4 % currently work-disabled and an additional 15.5 % having experienced disability within the past year.

**Discussion and conclusions:**

This prospective study highlights the significant multidimensional burden of low back pain among neurosurgical outpatients, characterized by substantial functional disability, high prevalence of depression, frequent erectile dysfunction, and marked occupational impairment. Our results underscore the critical need for routine psychological screening, proactive sexual health assessment, and targeted occupational rehabilitation within specialized neurosurgical care pathways. Implementing these comprehensive approaches may improve patient outcomes, support return to work, and mitigate potential socioeconomic impacts associated with degenerative lumbar spine disorders.

## Highlights

• **Heavy burden in lumbar disease:** High pain (NRS 7.0), severe disability (ODI 57), 42% depression, and 55% erectile dysfunction.
• Disability was independently predicted by higher PHQ-9 scores and older age in men, greater ED severity related to higher disability and older age.
• Surgery was associated with higher baseline ODI; working-age impact was marked (39 % currently work-disabled, +15.5 % in past year), supporting routine psychological/sexual-health screening and targeted occupational rehabilitation.

## 1 Introduction

Low back pain (LBP) is recognized globally as a prevalent cause of chronic disability, significantly impacting patients' quality of life, psychosocial well-being, and occupational productivity (Hartvigsen et al., 2018; Wu et al., 2012). Worldwide, approximately 7.5 % of individuals—over 500 million people—experience low back pain at any given time, with its societal burden progressively increasing, particularly in high-income countries due to aging populations (Hartvigsen et al., 2018). Epidemiological studies from Germany indicate that over 60 % of adults report lumbar pain annually, and approximately 15 % suffer from chronic pain, leading to substantial healthcare expenditures and productivity losses, amounting to nearly 1 % of the national gross domestic product (von der Lippe et al., 2020; Wenig et al., 2009). While the majority of these patients receive conservative treatment in primary care settings, approximately 5–10 % of chronic lumbar pain patients require referral to specialized care, including neurosurgical evaluation (Grøndahl et al., 2024). Importantly, patients presenting to neurosurgical outpatient clinics represent a highly selected subgroup, specifically referred for consideration of surgical interventions such as spinal decompression or fusion procedures.

Demographic and socioeconomic factors significantly influence the onset, severity, and progression of LBP. Epidemiological data suggest higher prevalence rates among elderly individuals and women, likely attributable to age-related degenerative spinal changes and sex-specific psychosocial factors (Meucci et al., 2015; Wong et al., 2017). Socioeconomic disadvantages, including lower educational attainment, physically demanding occupations, and restricted healthcare access, markedly elevate the risk of chronicity and associated functional impairment in patients with lumbar pain (Shmagel et al., 2016). Notably, manual laborers are disproportionately affected by lumbar pain and related disabilities, frequently experiencing prolonged occupational impairment and severe economic consequences (Meucci et al., 2015).

Beyond physical limitations, LBP is frequently associated with significant psychosocial comorbidities, including elevated rates of depression and erectile dysfunction. Depressive symptoms commonly coexist with chronic pain, exacerbating pain perception and impeding recovery, thus perpetuating a detrimental cycle of psychological distress and chronic disability (Ogliari et al., 2023; Pinheiro et al., 2016). Additionally, chronic lumbar pain is increasingly recognized as being linked to erectile dysfunction; however, this critical aspect of patients' quality of life is often overlooked in clinical practice (Berg et al., 2009; Nikoobakht et al., 2014). Although extensive literature exists documenting these multidimensional impacts, most studies have examined these associations individually, rather than collectively, particularly within specialized outpatient settings.

To address this critical knowledge gap, we conducted a prospective cohort study examining demographic, clinical, and psychosocial characteristics in a series of 247 consecutive patients presenting with LBP at the outpatient department of Neurosurgery, University Hospital Heidelberg. The primary aim of our study was to explore potential associations between lumbar pain severity and functional disability, severity of depressive symptoms, erectile dysfunction (ED), and sociodemographic parameters.

## 2 Methods

### 2.1 Study design and participants

This prospective cross sectional study, utilizing a **cross-sectional analysis of baseline data**, was conducted at the neurosurgical outpatient clinic of Heidelberg University Hospital (UKHD) between February 1 and June 30, 2025. We systematically screened all consecutive adult patients (≥18 years) presenting with LBP attributed to degenerative spinal conditions, defined as symptomatic disorders resulting from degenerative disc disease, spinal canal stenosis, facet joint arthrosis, or spondylolisthesis, excluding acute trauma, spinal malignancies, infectious or inflammatory spinal pathologies, or significant cognitive impairment precluding questionnaire completion. LBP was defined as pain localized to the lumbar region, with or without radicular symptoms. Exclusion criteria were acute trauma, spinal malignancies, infectious or inflammatory spinal pathologies, or significant cognitive impairment precluding questionnaire completion. **General medical comorbidities and a history of prior lumbar surgery were not exclusion criteria.** While a specific pain duration was not a primary inclusion criterion, the focus on degenerative spinal conditions inherently selected for a patient population with predominantly chronic symptoms, as reflected in our cohort. Informed consent for clinical documentation and electronic patient-reported outcome measures (PROMs) as part of routine clinical practice was obtained for eligible patients. The study was conducted in accordance with the ethical principles outlined in the Declaration of Helsinki. Approval for the scientific evaluation of those data was obtained from the Ethics Committee of the Medical Faculty, Heidelberg University (approval number: S-096/2025).

### 2.2 Baseline data collection and questionnaires

Baseline demographic data collected included age, sex, nationality, marital status, occupation, and insurance status. Clinical characteristics documented were body mass index (BMI), smoking status, alcohol consumption, physical activity levels, prior conservative or surgical treatments, pain duration, and functional impairments. **For sociodemographic analysis,** marital status was categorized as single, married, divorced, or widowed. Occupational status was recorded based on patient self-report (e.g., 'mechanic,' 'retired,' 'office worker'). 'Current work disability' was defined as a patient's self-report of being unable to work at their regular occupation at the time of presentation due to their lumbar symptoms. Patients were classified into surgical and non-surgical groups according to their clinical pathway following initial outpatient assessment: those who subsequently underwent neurosurgical intervention (decompression surgery) due to clinically and radiologically confirmed lumbar disc herniation or spinal canal stenosis were assigned to the surgical group, while patients managed conservatively without surgery formed the non-surgical group. Standardized, validated PROMs were electronically administered at baseline and included assessments of pain intensity (Numeric Rating Scale [NRS]), functional disability (Oswestry Disability Index [ODI]), depressive symptoms (Patient Health Questionnaire-9 [PHQ-9]), and erectile dysfunction (International Index of Erectile Function-5 [IIEF-5], administered to male patients). Proms are depicted in supplemantary Table. The selection of these specific PROMs was intended to capture the multidimensional burden of LBP, directly corresponding to the study's primary aims of assessing physical function, psychological comorbidity, and sexual health. All questionnaires were scored and interpreted according to their original, validated protocols, and standard cut-off points were used to categorize severity. Validated, language-specific versions of the PROM questionnaires were available in multiple languages (German, English, Turkish, Arabic, Polish, Romanian, and Italian) to facilitate inclusivity and patient comprehension. The standardized, validated versions of these questionnaires were administered without any modification or adaptation to content or scoring. Patients completed these questionnaires on institutional tablets (iPads) or their personal electronic devices (smartphones, tablets, laptops, or desktop computers).

### 2.3 IT infrastructure and data security

The electronic data capture (EDC) system employed in this study (MyEDC) was fully compliant with institutional IT security standards. It operated as a secure, web-based platform accessible from any personal device with internet connectivity and standard web browsers (HTML5 compatibility). The EDC platform included automated completeness checks, which prompted patients to fill in any required fields that were left blank before final submission, thereby minimizing missing data. No specialized software or applications needed to be installed on patients’ personal devices, ensuring ease of access. Data security was maintained through multiple firewall layers, reverse proxy servers, secure data encryption, strict access controls, and rigorous internal data management procedures approved by the institutional Information Security Office at UKHD. Patient data were uploaded into the institutional electronic health record system (EHR; i.s.h.med, Cerner Inc.).

### 2.4 Statistical analysis

**A formal a priori sample size calculation was not performed.** This study was designed as an observational cohort study to prospectively enroll all consecutive, eligible patients presenting within the defined 5-month study period, aiming to capture a representative sample of this specialized outpatient population. For all continuous variables (age, BMI, NRS, ODI, PHQ-9, IIEF-5), distributional assumptions were assessed using **Shapiro–Wilk tests** and **visual inspection of histograms and Q–Q plots**. Because PROM scores showed moderate deviations from normality and ceiling effects, bivariate analyses were performed using **non-parametric methods**: correlations between continuous variables were calculated using **Spearman's rank correlation coefficients (ρ)**; group comparisons of continuous outcomes across two categories used **Mann–Whitney U tests**, and across three or more categories **Kruskal–Wallis tests**. Comparisons between categorical variables were conducted using **χ^2^ tests** or **Fisher's exact tests** where expected cell frequencies were <5. To identify **independent predictors of higher burden**, we performed multivariable regression analyses. First, linear regression analyses explored associations between depression severity (PHQ-9), pain intensity (NRS), and disability (ODI). Subsequently, **multivariable linear regression models** were fitted with **ODI scores** and, in male patients, **IIEF-5 scores** as dependent variables in order to identify independent predictors of functional disability and erectile dysfunction severity, respectively. Candidate predictors in these models included sociodemographic factors (age, sex, insurance status, marital status, current or recent work disability), lifestyle factors (smoking status, alcohol consumption), clinical characteristics (BMI, NRS pain scores), and psychological assessment (PHQ-9). In addition, a **multivariable logistic regression model** was constructed to examine factors associated with undergoing surgical treatment (yes/no). All variables of interest were entered simultaneously. For linear models, residual plots were inspected to confirm approximate normality and homoscedasticity; for the logistic model, linearity in the logit and absence of multicollinearity were assessed using standard diagnostics. Regression coefficients (B) with standard errors and p-values are reported for linear models, and odds ratios (OR) with 95 % confidence intervals (CI) for the logistic model. The significance threshold was set at **p < 0.05**. Variables with significant relationships with disability, surgical intervention, or erectile dysfunction were included, and arrows indicate the direction and sign (positive/negative) of these associations.

**Missing data handling** All analyses were based on **available data** for the respective variables. For each outcome (NRS, ODI, PHQ-9, IIEF-5), patients with missing values were excluded from that specific analysis; **no statistical imputation** was performed. Multivariable regression models were fitted as **complete-case analyses**, including only participants with non-missing data on the dependent variable and all covariates in the model. Consequently, denominators may vary across analyses; the exact sample size (n) for each comparison and each regression model is reported in the corresponding tables, figure legends, and supplementary tables.

All analyses were conducted using **R statistical software** (version 4.2.2, R Foundation for Statistical Computing, Vienna, Austria).

## 3 Results

### 3.1 Baseline patient characteristics

We prospectively enrolled 247 patients with chronic lumbar spine pain (mean age 60.4 ± 16.0 years, 53.8 % male, of whom 110 (44.5 %) underwent decompression surgery due to lumbar disc herniation or spinal canal stenosis. The cohort predominantly held statutory insurance (79.8 %), was mainly of German nationality (87.3 %), and was overweight or obese (mean BMI 28.2 ± 6.2 kg/m^2^). Most patients presented with chronic (>12 weeks) and severe pain (mean NRS 7.0 ± 2.2), with pronounced functional impairments, particularly affecting prolonged sitting or standing (60.9 %) and lifting or carrying (15.7 %). Notably, 33.5 % of patients reported being currently unable to work due to their lumbar pain, underscoring significant occupational impact. Among patients of working age (≤65 years, n = 142), 56 (39.4 %) were currently work-disabled, and an additional 22 (15.5 %) had experienced work disability within the past 12 months. The mean age of currently work-disabled patients was 48.5 ± 11.7 years (range: 20–65 years). Additional detailed baseline characteristics are presented in Table 1.

**Table 1:** Patient demographics and clinical characteristics (N = 247).

Characteristic	n (%) or mean ± SD (range)
**Age (years)**	60.4 ± 16.0 (20–92)
**Sex**	
Male	133 (53.8 %)
Female	114 (46.2 %)
**Insurance Status**	
Statutory	190 (79.8 %)
Private	45 (18.9 %)
Self-paying	3 (1.3 %)
**Occupational Categories**	
Retired/Pensioner	38 (16.2 %)
Manual & Skilled Labor	6 (2.6 %)
Healthcare & Social	4 (1.7 %)
Sedentary Office Job	2 (0.9 %)
Unemployed	2 (0.9 %)
Service Industry	1 (0.4 %)
Other/Unknown	182 (77.4 %)
**Nationality**	
German	207 (87.3 %)
Turkish	3 (1.3 %)
Polish	3 (1.3 %)
Ukrainian	3 (1.3 %)
Kosovar	2 (0.8 %)
Romanian	2 (0.8 %)
Serbian	2 (0.8 %)
Syrian	2 (0.8 %)
Other	13 (5.3 %)
**Marital Status**	
Married/Partnership	163 (68.5 %)
Single	48 (20.2 %)
Divorced/Separated	27 (11.3 %)
**Body Mass Index (BMI)**	28.2 ± 6.2 (15.3–59.9)
**Pain Duration**	
>12 weeks	169 (71.9 %)
6–12 weeks	33 (14.0 %)
<6 weeks	33 (14.0 %)
**Pain Score (NRS)**	7.0 ± 2.2 (3–10)
**Prior Treatments**	
Physiotherapy	84 (37.8 %)
Pain Medication	68 (30.6 %)
Lumbar Spine Surgery	44 (19.8 %)
Infiltration/Osteopathy	26 (11.7 %)
**Smoking Status**	
Never smoked	106 (44.7 %)
Former smoker	77 (32.5 %)
Active smoker	54 (22.8 %)
**Alcohol Consumption**	
Never	98 (41.7 %)
1–2 times per week	121 (51.5 %)
Daily	16 (6.8 %)
**Physical Activity**	
Rarely	130 (54.9 %)
1–2 times per week	56 (23.6 %)
>2 times per week	51 (21.5 %)
**Mobility Aids**	
None	160 (69.0 %)
Walking aids (crutches, rollator)	62 (26.7 %)
Corset	10 (4.3 %)
**Functional Impairments (daily/work)**	
Difficulty prolonged standing/sitting	140 (60.9 %)
Difficulty lifting/carrying	36 (15.7 %)
Difficulty climbing stairs	29 (12.6 %)
Impairment in leisure/hobbies/sports	14 (6.1 %)
Impairment in household tasks	11 (4.8 %)
**Work Disability**	
No current disability	127 (56.7 %)
Currently disabled	75 (33.5 %)
Disabled within last 12 months	22 (9.8 %)

### 3.2 Associations of demographic and lifestyle factors with pain intensity and disability

Correlation analyses showed no significant associations between age or BMI and either pain intensity (NRS: age r = −0.012, p = 0.860; BMI r = 0.002, p = 0.980) or functional disability (ODI: age r = 0.053, p = 0.430; BMI r = 0.061, p = 0.360). Group comparisons revealed no significant differences in NRS or ODI scores based on sex (NRS p = 0.820; ODI p = 0.970), marital status (NRS p = 0.950; ODI p = 0.480), or insurance type (NRS p = 0.750; ODI p = 0.640). However, patients actively smoking reported significantly higher functional disability (ODI, p = 0.041). Alcohol consumption was significantly associated with both higher pain intensity (NRS, p = 0.006) and increased functional disability (ODI, p = 0.007), particularly among daily alcohol users. Furthermore, individuals reporting current or recent (within last 12 months) occupational disability exhibited significantly higher pain intensity (NRS, p = 0.029) and greater functional disability (ODI, p = 0.025). Detailed results of the associations between demographic and lifestyle factors with pain intensity and disability are summarized in Table 2.

**Table 2:** Associations of demographic and lifestyle factors with pain intensity (NRS) and functional disability (ODI).

Variable	Group	NRS Score (Mean ± SD)	p-value NRS	ODI Score (Mean ± SD)	p-value ODI
**Sex**	Female	2.84 ± 1.60	0.820	57.95 ± 16.74	0.970
	Male	2.88 ± 1.51		58.29 ± 17.69	
**Insurance Status**	Statutory	2.79 ± 1.64	0.750	57.72 ± 16.54	0.640
	Private	2.58 ± 1.57		58.86 ± 16.72	
	Self-paying	2.67 ± 2.89		50.56 ± 9.48	
**Marital Status**	Single	2.75 ± 1.68	0.950	59.20 ± 15.15	0.480
	Married/Partnership	2.74 ± 1.65		58.17 ± 16.51	
	Divorced/Separated	2.63 ± 1.57		54.23 ± 18.21	
**Smoking Status**	Never	2.33 ± 1.59	0.110	52.96 ± 16.19	0.041
	Former	2.42 ± 1.59		54.38 ± 15.26	
	Active	2.68 ± 1.72		60.10 ± 17.91	
**Alcohol Consumption**	Never	2.40 ± 1.62	0.006	57.81 ± 15.01	0.007
	1–2x/week	2.56 ± 1.41		55.11 ± 17.20	
	Daily	3.10 ± 1.62		58.18 ± 17.91	
**Work Disability**	None	2.32 ± 1.73	0.029	62.24 ± 14.70	0.025
	Current	2.86 ± 1.55		59.84 ± 20.05	
	Last 12 months	2.92 ± 1.57		55.59 ± 16.09	

**Abbreviations:** NRS, Numeric Rating Scale (pain intensity, 0–10); ODI, Oswestry Disability Index (0–100). *Group comparisons performed using Kruskal–Wallis or Mann–Whitney U tests as appropriate.*

### 3.3 Patient-Reported Outcomes (PROMs)

The mean Oswestry Disability Index (ODI) score was high (57.4 ± 16.5), with most patients (82.2 %) classified as having severe to exaggerated disability. Depression, assessed via PHQ-9, was prevalent, with nearly half (42.2 %) reporting moderate-to-severe symptoms (mean 8.5 ± 5.8). Among male patients, erectile dysfunction (ED) was notably common (mean IIEF-5: 16.5 ± 9.1), affecting over half (55 %) at moderate-to-severe levels. Detailed distributions for these outcomes are summarized in Table 3. Detailed distributions of patient-reported outcomes (ODI, PHQ-9, and IIEF-5) are provided in Supplementary Fig. 1–3 (see Table 4).

**Table 3:** Patient-reported outcome measures (PROMs) of disability, depression, and erectile dysfunction.

Outcome Measure	Mean ± SD (range) or n (%)
**Oswestry Disability Index (ODI)**	
ODI total score	57.4 ± 16.5 (11.7–100)
Minimal disability	3 (1.3 %)
Moderate disability	39 (16.5 %)
Severe disability	93 (39.4 %)
Very severe (crippled)	83 (35.2 %)
Bed-bound or exaggerated symptoms	18 (7.6 %)
**Depression Severity (PHQ-9)**	
PHQ-9 total score	8.5 ± 5.8 (0–25)
Minimal depression (0–4)	70 (29.5 %)
Mild depression (Grøndahl et al., 2024; Meucci et al., 2015; Wong et al., 2017; Shmagel et al., 2016; Ogliari et al., 2023)	67 (28.3 %)
Moderate depression (Pinheiro et al., 2016; Berg et al., 2009; Nikoobakht et al., 2014; Benz et al., 2021; Mathieu et al., 2024)	58 (24.5 %)
Moderately severe depression (Brooks et al., 2013; Heuch et al., 2024; Burnham et al., 2022; Viniol et al., 2013; Lurie et al., 2015)	36 (15.2 %)
Severe depression (≥20)	6 (2.5 %)
**Erectile Dysfunction (IIEF-5; N=111 males)**	
IIEF-5 total score	16.5 ± 9.1 (0–30)
No erectile dysfunction	26 (23.4 %)
Mild erectile dysfunction	11 (9.9 %)
Mild-to-moderate erectile dysfunction	14 (12.6 %)
Moderate erectile dysfunction	21 (18.9 %)
Severe erectile dysfunction	39 (35.1 %)

**Abbreviations:** ODI = Oswestry Disability Index; PHQ-9 = Patient Health Questionnaire-9; IIEF-5 = International Index of Erectile Function. Higher ODI scores indicate greater functional disability; higher PHQ-9 scores indicate greater depressive severity; lower IIEF-5 scores indicate greater severity of erectile dysfunction.

**Table 4:** Comparison of Patient-Reported Outcomes by Surgical vs. Non-surgical Groups.

Outcome measure	Group	N	Mean ± SD	p-value
**Functional Disability (ODI)**	No Surgery	123	54.61 ± 16.46	**0.001**
	Surgery	108	61.47 ± 15.67	
**Depression (PHQ-9)**	No Surgery	122	9.29 ± 6.26	0.076
	Surgery	110	7.73 ± 5.24	
**Erectile Dysfunction (IIEF-5, males)**	No Surgery	32	19.81 ± 8.83	**0.034**
	Surgery	36	14.61 ± 9.50	

**ODI:** Oswestry Disability Index; **PHQ-9:** Patient Health Questionnaire-9; **IIEF-5:** International Index of Erectile Function-5. Lower IIEF-5 scores indicate greater erectile dysfunction severity.

**Note:** p-values were obtained from Mann–Whitney U-tests; significant p-values (p < 0.05) are in bold.

### 3.4 Associations between pain intensity, disability, and depression severity

**Fig. 1:**
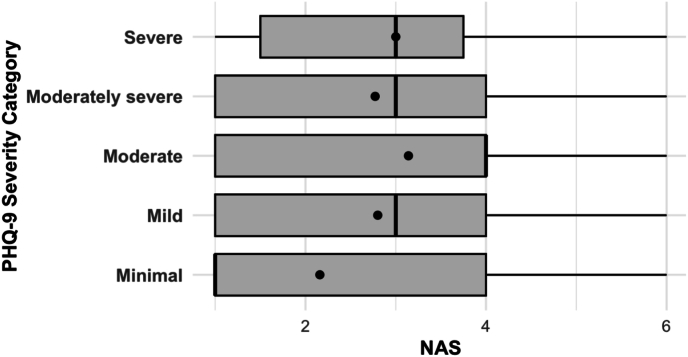
**Pain intensity (NRS scores) by depression severity (PHQ-9 categories) in patients with lumbar pain**. Horizontal boxplots show the distribution of Numeric Rating Scale (NRS) pain scores across PHQ-9 depression severity categories. Higher depression severity was associated with higher pain intensity (overall p = 0.014, Kruskal-Wallis test). Boxes represent interquartile ranges, lines within boxes indicate medians, dots represent means, and whiskers represent the data ranges.

**Fig. 2:**
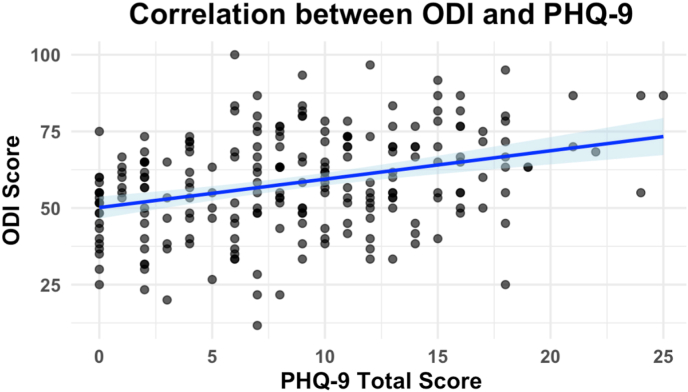
**Correlation between functional disability (ODI) and depression severity (PHQ-9) in patients with lumbar pain**. Scatter plot showing a positive correlation between Oswestry Disability Index (ODI) scores and Patient Health Questionnaire-9 (PHQ-9) total scores, indicating that greater disability due to lumbar pain is associated with more severe depressive symptoms. The blue line represents the regression line with a 95 % confidence interval.

Pain intensity (NRS) significantly increased with greater depression severity (PHQ-9 categories), with the highest NRS scores reported by patients in the moderate depression group (7.4 ± 1.8, overall p = 0.014; Fig. 1). Further, correlation analysis revealed a significant positive relationship between PHQ-9 total scores and NRS scores (Spearman's r = 0.184, p = 0.005). Similarly, functional disability (ODI) demonstrated a significant progressive increase across worsening depression severity categories, rising from minimal depression (52.01 ± 13.0) to severe depression (75.56 ± 13.0, overall p < 0.001). A robust correlation between PHQ-9 total scores and ODI scores was observed (Spearman's r = 0.319, p < 0.001; Fig. 2), emphasizing the strong interrelationship between depression and functional impairment in chronic lumbar pain patients. Supplementary Table 1 displayes pain Intensity (NRS) and functional Disability (ODI) by Depression Severity (PHQ-9 Categories). In the refitted multivariable linear model (parsimonious covariates: age, PHQ-9, surgery, any work-disability, insurance; complete-case analysis), PHQ-9 remained independently associated with higher disability (B = 1.27; 95 % CI 0.90–1.64; p < 0.001). There was no evidence that this association differed by sex (no PHQ-9× sex interaction).

Association Between Erectile Dysfunction and Functional Disability.

**Fig. 3:**
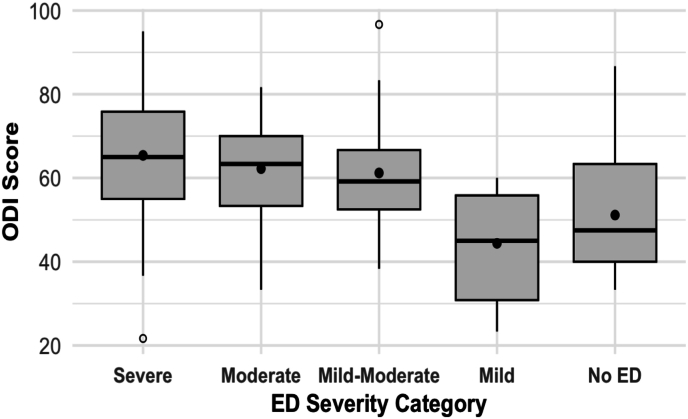
**Association between erectile dysfunction (ED) severity and functional disability (ODI) in male patients with lumbar pain (N=111).** Boxplot illustrating the distribution of Oswestry Disability Index (ODI) scores across ED severity categories (defined by IIEF-5). Higher ED severity is significantly associated with increased lumbar-related functional disability (p < 0.001, Kruskal-Wallis test). Boxes represent interquartile ranges (IQR, 25th–75th percentile), lines within boxes indicate medians, means are shown as dots, whiskers extend to 1.5 × IQR, and outliers are shown as open circles.

Among the 133 male patients in the cohort, 111 (83.5 %) completed the IIEF-5 questionnaire and were included in the sexual function analysis. The remaining 22 (16.5 %) male patients did not complete this specific questionnaire and were thus excluded from this sub-analysis. Among male patients, erectile dysfunction (ED) severity significantly correlated with functional disability (ODI), as measured by the IIEF-5 questionnaire (overall p < 0.001, Suppl. Table 2). Patients reporting severe ED demonstrated the highest ODI scores (65.38 ± 15.62), followed closely by moderate ED (62.22 ± 13.35) and mild-to-moderate ED (61.19 ± 15.84). Patients without ED or with only mild ED exhibited notably lower disability scores (51.15 ± 14.55 and 44.39 ± 13.73, respectively). Fig. 3 demonstrates the significant relationship between erectile dysfunction severity and increased functional disability (ODI scores) among male patients. In multivariable analysis among men (parsimonious covariates as above), worse erectile function (lower IIEF-5) was independently associated with older age (B = −0.25; 95 % CI -0.32 to −0.18; p < 0.001) and higher ODI (B = −0.15; 95 % CI -0.21 to −0.09; p < 0.001), while PHQ-9 and surgery status were not significant.

### 3.5 Multivariate analyses of predictors associated with functional disability, erectile dysfunction, and surgical treatment

**Fig. 4:**
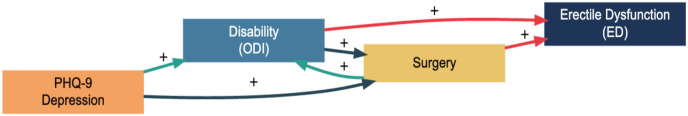
***Conceptual framework of hypothesised relationships between sociodemographic, clinical and psychosocial factors in neurosurgical low back pain patients***. Arrows labeled '+' indicate positive associations (higher values predict higher outcomes or greater impairment). Depression severity positively predicts disability and likelihood of surgery, while higher disability and indication for surgery independently predict worse erectile function.

The multivariate regression models identified significant predictors of functional disability (ODI), erectile dysfunction (IIEF-5), and the likelihood of undergoing surgical treatment. At baseline, patients subsequently selected for surgical treatment had significantly higher functional disability (ODI scores: mean ± SD 61.47 ± 15.67 vs. 54.61 ± 16.46, p = 0.001) and worse erectile function among males (IIEF-5 scores: 14.61 ± 9.50 vs. 19.81 ± 8.83, p = 0.034) compared to patients managed non-surgically. Using prespecified parsimonious covariate sets and complete-case analyses, the refitted models yielded the following independent associations (full estimates in Supplementary Tables). For ODI (linear regression), higher PHQ-9 (B = 1.27; 95 % CI 0.90–1.64; p < 0.001), undergoing surgery (B = 10.46; 95 % CI 5.95–14.98; p < 0.001), any current/recent work-disability (B = 9.56; 95 % CI 5.09–14.03; p < 0.001), and older age (B = 0.19 per year; 95 % CI 0.04–0.33; p = 0.010) were associated with greater disability, whereas insurance status was not. In the men-only IIEF-5 model, older age (B = −0.25; 95 % CI -0.32 to −0.18; p < 0.001) and higher ODI (B = −0.15; 95 % CI -0.21 to −0.09; p < 0.001) were associated with worse erectile function; PHQ-9 and surgery were not significant. For surgery (logistic regression), higher baseline ODI (OR = 1.04 per point; 95 % CI 1.02–1.06; p < 0.001) and private insurance (OR = 6.40; 95 % CI 2.40–17.07; p < 0.001) were associated with undergoing decompression, while higher PHQ-9 was associated with a lower likelihood (OR = 0.92 per point; 95 % CI 0.87–0.98; p = 0.005); age and work-disability were not significant after adjustment. Model diagnostics were satisfactory (logistic AUC ≈ 0.78; Nagelkerke R^2^ ≈ 0.28; all VIFs <1.3). (Suppl. Table 5). A conceptual framework is presented in Fig. 4.

## 4 Discussion

To our knowledge, this is the first prospective study to comprehensively assess the interplay between functional impairment, depression, erectile dysfunction (ED), and sociodemographic factors in a neurosurgical outpatient cohort with low back pain (LBP). In our large cohort of 247 predominantly older (mean age 60.4 years), male, and highly disabled patients (mean ODI 57.4; 82.2 % severe or worse), we observed remarkably high rates of moderate-to-severe depressive symptoms (42.2 %) and ED (55 %), underscoring substantial but frequently overlooked psychosocial burdens. Additionally, our cohort demonstrated notable obesity (mean BMI 28.2 ± 6.2 kg/m^2^), an important finding due to its known association with multiple medical comorbidities, including diabetes, cardiovascular diseases, hypertension, stroke, and recurrent musculoskeletal disorders, and because obesity represents a potentially modifiable risk factor through targeted therapeutic interventions.Importantly, multivariate regression revealed depression severity (PHQ-9) as a significant independent predictor of functional disability, alongside older age and surgical treatment. Furthermore, older age and higher functional disability significantly were associated with worse erectile function. Although age and BMI did not directly predict pain intensity, lifestyle factors including increased alcohol and tobacco use emerged as relevant correlates, potentially reflecting maladaptive coping mechanisms. Multivariate logistic regression demonstrated that higher functional disability (ODI) and private insurance status significantly increased the likelihood of surgical intervention, while former smokers were less likely to undergo surgery. Additionally, the substantial prevalence of occupational disability (33.5 %) emphasizes significant socioeconomic and healthcare implications. Collectively, these findings highlight the necessity of systematically addressing depression, erectile dysfunction, obesity and occupational factors within comprehensive neurosurgical management strategies for patients with low back pain.

### 4.1 Patient characteristics and clinical profile in patients with LBP

Our neurosurgical outpatient LBP cohort (mean age ∼60 years) was notably older and more often male than typical non-surgical LBP cohorts. For example, a Swiss interdisciplinary pain clinic study reported a mean age of 48 with ∼59 % female patients (Benz et al., 2021), whereas our sample was predominantly male despite women generally having higher LBP prevalence (Mathieu et al., 2024). This likely reflects referral patterns; degenerative spine conditions increase with age and commonly prompt surgical evaluation. Interestingly, neither age nor body mass index (mean BMI ∼28) correlated with pain intensity or disability in our cohort. Prior research similarly found that BMI alone is not a strong determinant of LBP severity or outcomes (Brooks et al., 2013), even though obesity elevates LBP risk at the population level (Heuch et al., 2024). Additionally we did find notably low levels of physical activity (54.9 % reporting minimal or rare physical activity) as well as approximately 20 % of patients in our cohort had already undergone prior spine surgery. The relatively high proportion of repeat surgeries may be attributable to the low levels of physical activity and high BMI observed in our patient population. Therefore, it is imperative to develop and implement targeted secondary prevention strategies, including structured rehabilitation and psychosocial interventions, to reduce recurrence and minimize the necessity for repeated surgical interventions.

Pain and functional limitation were tightly linked: higher NRS pain scores corresponded to worse ODI scores, as expected (Burnham et al., 2022). The severity of symptoms in our patients – mean NRS ∼7/10 and ODI ∼57 (with >80 % classified as severe or bedridden) – appears higher than that reported in general primary care LBP samples (Benz et al., 2021). This aligns with the notion that neurosurgical clinics see a more refractory, high-disability subset of LBP patients (e.g. an older degenerative-pain subgroup with low work participation) (Viniol et al., 2013). Our multivariate analyses further reinforced this finding, demonstrating that higher disability scores (ODI) significantly predicted an increased likelihood of undergoing surgery (OR = 1.04, p = 0.032), consistent with clinical practice and prior studies indicating that greater functional impairment often drives surgical decision-making in spinal care (Wenig et al., 2009; Lurie et al., 2015). Furthermore, the average ODI of 57 and the proportion classified as “severe” or “bedbound” (>80 %) exceed values reported for general outpatient LBP (typically ODI ≈ 35–40), underscoring the high burden carried by patients who reach neurosurgical care (Hartvigsen et al., 2018). We also observed a greater prevalence of smoking (and to some extent alcohol use) among those with more intense pain, suggesting these patients may engage in maladaptive coping. Alternatively, smoking might directly contribute to lower physical activity levels, reduced muscle mass, and consequently increased pain severity. Indeed, studies indicate many individuals with chronic pain use tobacco or alcohol as a form of self-medication, despite its risks (Schaffer et al., 2023). Prior evidence suggests that up to one-third of people with persistent musculoskeletal pain self-medicate with nicotine or alcohol to transiently blunt nociception or negative affect (Bonanni et al., 2022). Cross-sectional studies from Germany likewise demonstrates a dose–response relationship between back-pain intensity and risk‐behaviour profiles, even after adjustment for socioeconomic status (Schmidt et al., 2011). Our data reinforce these observations and highlight the need for systematic screening of substance use in spine clinics, as continued nicotine or alcohol exposure is linked to poorer long-term pain trajectories and reduced treatment response (Pinheiro et al., 2016). Interestingly, former smokers in our cohort exhibited significantly lower odds of undergoing surgery, diverging from epidemiological data indicating smokers typically have higher rates of spinal surgery due to accelerated spinal degeneration (Shiri et al., 2017). This unexpected result might reflect patient selection or behavioral differences specific to our tertiary outpatient clinic and warrants further exploration.

### 4.2 Occupational disability, economic impact, and influence of insurance status in patients with low back pain

In our cohort, the economic and occupational impact is further highlighted by the notably high proportion of current (39.4 %) and recent (15.5 %) work disability specifically among patients of working age. This underscores the profound socioeconomic implications of chronic low back pain, particularly within the economically active population, highlighting the importance of targeted vocational rehabilitation interventions in this subgroup. Low back pain remains the leading cause of activity limitation, sick leave and lost productivity worldwide (Yang et al., 2023) and high rates of work disability are well documented in severe chronic LBP populations (Viniol et al., 2013). One-in-three participants was on current medical leave and another 10 % had been work-disabled within the preceding year—figures strikingly higher than the 11–18 % annual work-absence rates reported for community-dwelling Germans with LBP (Wenig et al., 2009)(. Lost productivity from back-related sick leave already costs Germany ≈ €16 billion annually (≈0.6 % GDP), while direct medical expenditures add a further €9–10 billion (von der Lippe et al., 2020). Our multivariate analyses further support these findings by demonstrating a significant association between work disability status and functional impairment (ODI). Patients reporting recent work disability (within the past 12 months) had significantly higher ODI scores, highlighting a reciprocal relationship wherein severe disability contributes to sustained work absence, further exacerbating disability and economic burden (OR = 1.04 per ODI point increase; p = 0.032). Extrapolating our disability rate to national estimates suggests the economic burden borne by patients severe enough to require tertiary consultation is disproportionately large. International meta-analyses confirm that once absence exceeds three months, return-to-work likelihood falls below 50 %, thus leading to a cycle of chronic pain and economic inactivity (Feuerstein et al., 2004).

Our study found that privately insured patients had a significantly higher likelihood of undergoing surgery, suggesting possible disparities in healthcare access or decision-making. Despite Germany's universal healthcare, privately insured individuals, though a minority, may receive faster access to specialists or quicker surgical approvals. Similar disparities based on insurance type have been documented internationally; for instance, a U.S. study noted faster surgical evaluations for privately insured patients (Lima et al.). In our context, the higher surgery rates among privately insured patients might reflect differences in healthcare pathways, patient expectations, or socioeconomic status rather than strictly clinical criteria. Given the limited specific data on insurance-related surgery rates in Germany, these findings highlight the need for further research on how insurance status influences clinical decision-making and patient outcomes.

### 4.3 Biopsychosocial integration

In line with the biopsychosocial model, our findings on socioeconomic factors highlight how non-biological elements modulate the LBP burden. We identified private insurance status as a powerful predictor of undergoing surgery (OR 22.39) and found that occupational disability was extensive (39.4 % among working-age patients). This supports the concept that LBP is not merely a biological state but a complex experience with a profound 'economic burden' that is inseparable from the patient's social context. As highlighted by De Simone et al. (2024) (De Simone et al., 2024), LBP is a leading global cause of disability, and socioeconomic factors can influence everything from pain perception and healthcare access to clinical outcomes. Our data strongly support this, suggesting that occupational status and insurance type are critical components of the "disability" construct in this population.

### 4.4 Depression and Functional Disability in low back pain: clinical Relevance and methodological considerations

A central finding from our prospective cohort was the notably high prevalence (42.2 %) of moderate-to-severe depressive symptoms. This prevalence significantly surpasses the rates observed in general populations (5–10 %) and aligns with elevated depression rates previously reported in specialized pain-management contexts. For instance, a retrospective analysis of administrative data from 120,000 chronic muscoskelettal pain patients in the United Kingdom reported depression in approximately 40 % of cases; however, this study was limited by reliance on clinical coding, likely underestimating actual symptom prevalence due to inconsistent provider documentation (Rayner et al., 2016). Similarly, a large multinational cross-sectional analysis of over 246,000 older adults with chronic pain found pooled depression rates approaching 35–45 %, yet relied on retrospective self-reporting, which can introduce recall bias and under-reporting (Ogliari et al., 2023). By contrast, our prospective data collection, utilizing validated instruments (PHQ-9), likely provides a more accurate and timely measure of the actual depressive burden. Importantly, our analysis demonstrated a significant positive correlation between depression severity and functional disability (ODI scores), corroborating previous prospective studies such as the systematic review by Pinheiro et al. (2016), which included data from 23 prospective cohorts (total N = 23,109) and consistently found depression to be a strong independent predictor of greater disability. Our multivariate linear regression analysis further reinforced this association, clearly identifying depression severity (PHQ-9) as a significant independent predictor of higher ODI scores even after adjusting for other relevant demographic and clinical variables (age, sex, BMI, smoking, alcohol use, and surgical status). Unlike some previous studies that were limited by their retrospective nature or absence of multivariate adjustments, our regression analyses controlled for multiple confounding factors, thus providing robust support for the depression-disability association. However, our assessment of depressive symptoms relied solely on the PHQ-9 questionnaire, without formal psychiatric evaluation, which may somewhat limit the clinical interpretation of these findings. These findings potentially highlight that targeted psychological interventions to address depression may be critical to effectively reduce functional impairment in patients with chronic low back pain, regardless of demographic characteristics.

Depression and Functional Disability.

Our findings on the psychological burden also warrant a broader comparison. The prevalence of moderate-to-severe depressive symptoms in our cohort (42.2 %) is notably high. For instance, it is more than double the 20.19 % pooled prevalence reported in a recent 2025 systematic review and meta-analysis of patients with lumbar degenerative disc disease, and significantly higher than the 25.00 % prevalence of depressive symptoms found in a large 2022 study of over 1100 chronic low back pain patients in China (Scrofani et al., 2025). This stark difference likely underscores the highly selected nature of our tertiary neurosurgical outpatient population. Patients referred to our clinic often represent a 'refractory, high-disability subset' who have failed primary care management, and our data suggest this burden is compounded by severe, and often unaddressed, psychological distress.

Erectile dysfunction in Low Back Pain.

Moreover, our study uniquely provided prospective data on ED—an important but often overlooked outcome in neurosurgical spine care. Moderate-to-severe ED was reported by 55 % of male participants, a notably high prevalence consistent with specialized studies such as that by Nikoobakht et al. (2020), who reported ED rates of approximately 60 % in 120 Iranian patients with chronic lumbar pain (Nikoobakht et al., 2014). However, previous studies examining erectile dysfunction in lumbar pain patients typically employed retrospective methodologies or administrative claims data, likely underestimating the true extent due to stigma-related under-reporting or inadequate documentation (Berg et al., 2009; Pakpour et al., 2018). For instance, Berg et al. (2009), in a retrospective survey of 1032 lumbar disc herniation patients, acknowledged under-reporting of erectile dysfunction due to the sensitive nature of the topic and methodological constraints such as recall bias. Our prospective approach using a validated, confidential questionnaire (IIEF-5) likely overcame many such limitations, enhancing accuracy and completeness in reporting erectile dysfunction. Furthermore, we found a significant positive correlation between ED severity and ODI scores, supporting prior literature linking functional impairment and erectile dysfunction (Nikoobakht et al., 2014; Pakpour et al., 2018). Multivariate regression analysis further clarified these associations, identifying both older and greater functional disability as significant independent predictors of worse erectile function. Interestingly, depressive symptoms (PHQ-9) and surgical status did not independently predict ED in our cohort, diverging somewhat from previous research that commonly identifies depression as a contributing factor to erectile dysfunction in chronic pain populations (Vanti et al., 2023). This discrepancy could reflect differences in cohort composition, sample size, or depression assessment methods, and underscores the need for continued research into psychological contributors to sexual health in low back pain patients.

### 4.5 Neurosurgical context & implications

From a neurosurgical management perspective, our findings have direct, practical implications. The high prevalence of depression (42.2 %) and erectile dysfunction (55 %) in our cohort identifies a population with significant psychosocial risk factors, which are known to negatively correlate with surgical satisfaction and outcomes. This mandates that multidisciplinary screening—including psychological and sexual health assessment—should be a routine component of the neurosurgical workup, not an afterthought. For patients deemed surgical candidates, their high-risk psychosocial profile necessitates a management strategy that minimizes surgical burden and accelerates recovery. Recent meta-analyses, such as Scrofani et al. (2025), demonstrate that minimally invasive techniques like percutaneous transforaminal endoscopic discectomy (PTED) are associated with shorter hospital stays and fewer complications compared to traditional open discectomy (Scrofani et al., 2025). For a high-risk, highly disabled cohort like ours, the adoption of such minimally invasive approaches, where clinically appropriate, could be critical for facilitating faster rehabilitation and mitigating the socioeconomic impact of prolonged recovery.

### 4.6 Healthcare policy implications

Finally, our study raises important healthcare policy implications. The striking finding that private insurance was a powerful predictor of receiving surgery, independent of disability, points to potential socioeconomic disparities in care access. From a policy perspective, prevention strategies must not only focus on modifiable risk factors like obesity but also address the occupational factors that drive chronic disability. Furthermore, as new technologies like robotic-assisted minimally invasive surgery become available for degenerative spinal conditions (Choucha et al., 2025), policy-makers must ensure equitable access and robustly assess their cost-effectiveness. Our data suggest that without such considerations, access to advanced neurosurgical care could be increasingly dictated by insurance status rather than clinical need, further widening health disparities.

### 4.7 Limitations

This study has several limitations that should be considered. First, the data were collected from a single tertiary neurosurgical outpatient clinic, potentially limiting generalizability to broader chronic lumbar pain populations. Second, the cross-sectional design means there is a **complete absence of longitudinal follow-up or data on intervention outcomes**. This limitation prevents the determination of causal relationships … and we agree that longitudinal analyses would be necessary to confirm these findings and assess changes over time. Additionally, our reliance on patient-reported outcome measures, while standardized and validated, could introduce recall or response bias, particularly regarding sensitive topics such as erectile dysfunction or lifestyle habits. **Regarding the representativeness of our cohort and potential for bias,** our sample was predominantly **male (53.8 %)**, which, as previously noted, contrasts with general LBP populations but aligns with typical neurosurgical referral patterns. Therefore, our findings may be less generalizable to female-dominant primary care settings. In terms of socioeconomic status, our cohort largely held **statutory insurance (79.8 %)**, suggesting it is a broad cross-section of the general population rather than a specific 'lower-income' group. However, we did not collect detailed data on income, education level, or specific occupational categories (e.g., 'manual laborer'). The inability to analyze these specific factors, which are known to be associated with LBP, is a limitation of this study. Further limitations include the lack of detailed sociodemographic data, such as **patient-reported income, education level, and standardized occupational categories** (e.g., manual vs. sedentary labor). We also did not formally assess other key psychosocial variables like **anxiety or social** support, which could be important confounders in the relationship between pain, disability, and depression. Finally, potential confounders were not systematically evaluated. This is particularly relevant for the analysis of erectile dysfunction, as we did not collect data on key potential confounders such as antidepressant or other specific medication use, diabetes, peripheral vascular disease, or other comorbidities known to affect erectile function. These unmeasured variables may have significantly influenced the observed associations. An unexpected finding was the significantly lower likelihood of surgery among former smokers, contrasting with epidemiological data linking smoking to higher surgery rates due to accelerated disc degeneration and chronic pain. Possible explanations include selection bias, confounding patient characteristics such as age or health-conscious behaviors, or differences in surgeon-patient decision-making. This result emphasizes how sample-specific factors may influence observed associations and should not imply a protective effect of smoking cessation against surgery. Despite these limitations, the prospective nature, robust methodology, and comprehensive assessment of underreported psychosocial domains substantially enhance the validity and clinical relevance of our findings.

## 5 Conclusions

This prospective study highlights the significant burden of low back pain among patients in a neurosurgical outpatient setting, characterized by high levels of functional disability (82.2 %), frequent depression (42.2 %), and prevalent erectile dysfunction in males (55 %). ndependent predictors of increased disability included higher depression severity, older age, and undergoing surgical intervention. The notable proportion of occupationally disabled patients (approximately 40 % among working-age participants) further underscores the considerable socioeconomic implications of chronic lumbar disorders. These findings emphasize the necessity of routinely assessing psychological health, sexual function, occupational status, and modifiable lifestyle factors—such as obesity and physical inactivity—in specialized neurosurgical care. Such comprehensive and multidisciplinary approaches may help reduce symptom burden, facilitate return to work, and mitigate broader socioeconomic consequences associated with degenerative lumbar spine disorders.

## Consent to participate

Analysis of these routinely collected clinical data was covered under the institutional neurosurgical registry ethics approval (Ethics Committee of the Medical Faculty, Heidelberg University; approval number: S-096/2025), and thus separate informed consent for scientific evaluation was not required.

Data Material Availability The datasets generated during and/or analyzed during the current study are available from the corresponding author on reasonable request.

Ethics approval This study was approved by the Institutional Review Board of Heidelberg University (approval no. S-607/2023 and S-096/2025).

Authors’ contributions All authors contributed to the study conception and design. Data collection and analysis were performed by Pavlina Lenga, Sandro Krieg and Martin Dugas. The first draft of the manuscript was written by Pavlina Lenga. Sandro Krieg, Martin Dugas, Robin Fleige, Max Christian Blumenstock, Matthias Ganzinger and Sebastian Ille commented on previous versions of the manuscript. All authors read and approved the final manuscript.

## Consent for publication

No individual person's data were included in this study.

## Authors' information

Pavlina Lenga, Department of Neurosurgery and Institute of Medical Informatics, Heidelberg University Hospital, Heidelberg, Germany; Robin Fleige, Institute of Medical Informatics, Heidelberg University Hospital, Heidelberg, Germany; Max Christian Blumenstock, Institute of Medical Informatics, Heidelberg University Hospital, Heidelberg, Germany; Matthias Ganzinger, Institute of Medical Informatics, Heidelberg University Hospital, Heidelberg, Germany; Sebastian Ille, Department of Neurosurgery, Heidelberg University Hospital, Heidelberg, Germany; Sandro Krieg, Department of Neurosurgery, Heidelberg University Hospital, Heidelberg, Germany; Martin Dugas, Institute of Medical Informatics, Heidelberg University Hospital, Heidelberg, Germany.

## Declaration of conflicting interests

The author(s) declared no potential conflicts of interest with respect to the research, authorship, and/or publication of this article.
