# Supplementary material for: Multidimensional burden of low back pain: A prospective cross sectional study of patient-reported outcomes and sociodemographic factors at a tertiary neurosurgical center
Source: Brain Spine. 2025 Dec 13;6:105905. doi: 10.1016/j.bas.2025.105905 (PMC12794512; doi:10.1016/j.bas.2025.105905)
Supplement: Multimedia component 1 [file mmc1.docx]

**Supplementary Material**

**Supplemental Tables**

**A Supplementary Table. Overview of patient-reported outcome measures (PROMs) used in the study” was added as correctly recommended by the reviewer**

| **PROM (abbreviation)** | **Construct / domain** | **Items** | **Score range (direction)** | **Common interpretation categories*** |
| --- | --- | --- | --- | --- |
| Numeric Rating Scale (NRS-11) | Pain intensity (low back and/or leg pain) | 1 | 0–10 (0 = no pain, 10 = worst pain imaginable) | Often grouped as 0: no pain; 1–3: mild; 4–6: moderate; 7–10: severe (non-mandatory, used descriptively). |
| Oswestry Disability Index (ODI v2.1) | Back-specific functional disability | 10 | 0–100% (higher = worse disability) (1) | 0–20% minimal, 21–40% moderate, 41–60% severe, 61–80% crippling, 81–100% bed-bound/exaggeration. |
| Patient Health Questionnaire-9 (PHQ-9) | Depressive symptom severity | 9 | 0–27 (higher = more severe depression) (2) | 0–4 none/minimal, 5–9 mild, 10–14 moderate, 15–19 moderately severe, 20–27 severe. |
| International Index of Erectile Function-5 (IIEF-5) | Erectile function / erectile dysfunction | 5 | 5–25 (lower = more severe ED) (3) | 5–7 severe, 8–11 moderate, 12–16 mild-to-moderate, 17–21 mild, 22–25 no ED. |

1. Fairbank JC, Pynsent PB. The Oswestry Disability Index. Spine (Phila Pa 1976). 2000 Nov 15;25(22):2940–52; discussion 2952.

2. Kroenke K, Spitzer RL, Williams JB. The PHQ-9: validity of a brief depression severity measure. J Gen Intern Med. 2001 Sept;16(9):606–13.

3. Rosen RC, Cappelleri JC, Smith MD, Lipsky J, Peña BM. Development and evaluation of an abridged, 5-item version of the International Index of Erectile Function (IIEF-5) as a diagnostic tool for erectile dysfunction. Int J Impot Res. 1999 Dec;11(6):319–26.

**Table 1. Pain Intensity (NRS) and Functional Disability (ODI) by Depression Severity (PHQ-9 Categories)**

| **PHQ-9 Depression Severity** | **N** | **NRS Score (Mean ± SD)** | **ODI Score (Mean ± SD)** |
| --- | --- | --- | --- |
| Minimal | 69 | 2.16 ± 1.49 | 52.01 ± 13.0 |
| Mild | 65 | 2.77 ± 1.46 | 57.66 ± 18.0 |
| Moderate | 57 | 2.80 ± 1.56 | 59.21 ± 13.0 |
| Moderately severe | 35 | 3.00 ± 1.90 | 66.08 ± 15.0 |
| Severe | 6 | 3.14 ± 1.79 | 75.56 ± 13.0 |
| **Overall p-value** |  | **0.014** | **<0.001** |

**Abbreviations:** 3, Numeric Rating Scale (pain intensity, 0–10); ODI, Oswestry Disability Index (0–100); PHQ-9, Patient Health Questionnaire-9 (depression severity). *Statistical analysis: Kruskal-Wallis tests were used for comparisons across depression severity categories.*

**Table 2. Association Between Erectile Dysfunction (ED) Severity and Functional Disability (ODI)**

| **ED Severity Category** | **N** | **ODI Score (Mean ± SD)** |
| --- | --- | --- |
| No ED | 26 | 51.15 ± 14.55 |
| Mild ED | 11 | 44.39 ± 13.73 |
| Mild-to-moderate ED | 14 | 61.19 ± 15.84 |
| Moderate ED | 21 | 62.22 ± 13.35 |
| Severe ED | 39 | 65.38 ± 15.62 |
| **Overall p-value** |  | **<0.001** |

*ODI: Oswestry Disability Index (0–100), higher scores indicate greater disability. ED severity categorized according to the International Index of Erectile Function (IIEF-5). Statistical analysis by Kruskal-Wallis test.*

**Table 3. Multivariate Linear Regression Analysis Predicting Functional Disability (ODI)**

| Predictor | Estimate (B) | 95% CI | p-value |
| --- | --- | --- | --- |
| PHQ-9 (per point) | 1.27 | 0.90–1.64 | <0.001 |
| Surgery (yes) | 10.46 | 5.95–14.98 | <0.001 |
| Any work-disability (yes) | 9.56 | 5.09–14.03 | <0.001 |
| Age (per year) | 0.19 | 0.04–0.33 | 0.010 |
| Private insurance (vs non-private) | 1.96 | −4.15–8.06 | 0.528 |
| Model R² | 0.29 |  |  |

**ODI:** Oswestry Disability Index. Significant p-values (p<0.05) indicated in bold.

**Table 4. Multivariable Linear Regression Analysis Predicting Erectile Dysfunction (IIEF-5 Score)**

| Predictor | Estimate (B) | 95% CI | p-value |
| --- | --- | --- | --- |
| Age (per year) | −0.25 | −0.32 to −0.18 | <0.001 |
| ODI (per point) | −0.15 | −0.21 to −0.09 | <0.001 |
| PHQ-9 (per point) | −0.12 | −0.31–0.07 | 0.208 |
| Surgery (yes) | −0.25 | −2.51–2.01 | 0.829 |
| Private insurance (vs non-private) | 2.85 | 0.11–5.58 | 0.041 |
| Model R² | 0.52 |  |  |

**IIEF-5:** International Index of Erectile Function; **PHQ-9:** Patient Health Questionnaire-9; **ODI:** Oswestry Disability Index. **Note:** Lower IIEF-5 scores indicate greater erectile dysfunction severity. Significant results (p<0.05) are marked in bold.

## Table 5. Multivariable Logistic Regression Predicting Surgery (yes/no)

| Predictor | OR | 95% CI | p-value |
| --- | --- | --- | --- |
| ODI (per point) | 1.04 | 1.02–1.06 | <0.001 |
| PHQ-9 (per point) | 0.92 | 0.87–0.98 | 0.005 |
| Age (per year) | 1.01 | 0.99–1.03 | 0.605 |
| Any work-disability (yes) | 0.89 | 0.46–1.73 | 0.733 |
| Private insurance (vs non-private) | 6.40 | 2.40–17.07 | <0.001 |
| Model AUC | 0.78 |  |  |
| Nagelkerke R² | 0.28 |  |  |

Parsimonious model added (complete-case n = 215; 102 surgical / 113 non-surgical). **PHQ-9:** Patient Health Questionnaire-9; **ODI:** Oswestry Disability Index. Significant results (p<0.05) are marked in bold.

**
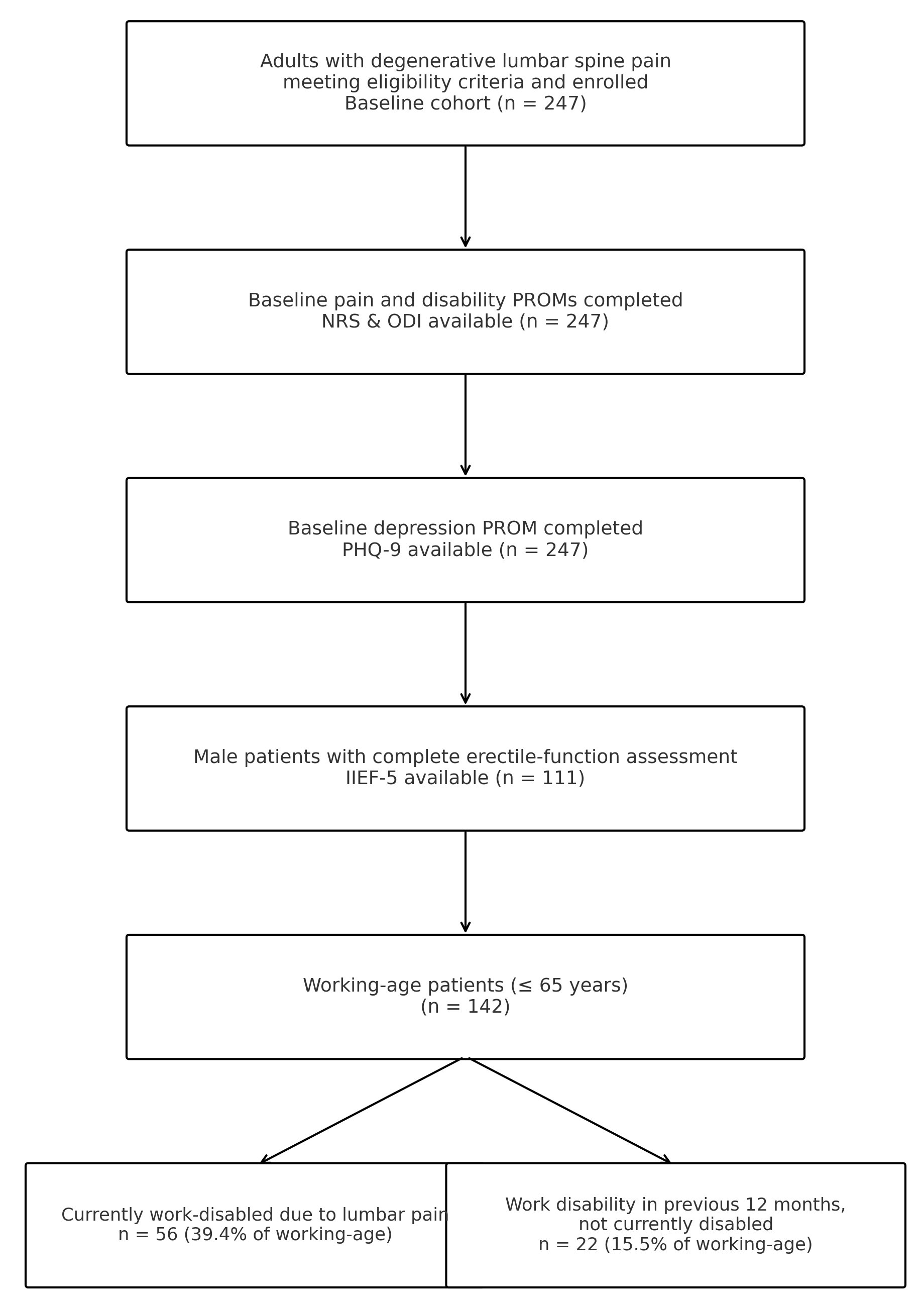
**

**Supplementary Figure 1. Participant flow diagram.**Flow of participants through the study from enrollment in the neurosurgical outpatient clinic to analytic subgroups. All 247 consecutively enrolled adults with degenerative lumbar spine pain who met the eligibility criteria and completed baseline patient-reported outcome measures (PROMs) formed the baseline cohort. The diagram shows the subgroups used in specific analyses: the full cohort with baseline pain and disability scores (NRS, ODI) and depression scores (PHQ-9), the subgroup of male patients with complete erectile-function data (IIEF-5, n = 111), and the working-age subgroup (≤65 years, n = 142) used for analyses of occupational disability. Differences in denominators across analyses therefore reflect predefined subgroups and questionnaire completion rather than loss to follow-up.

**Supplementary Figures: Patient-Reported Outcome Measures (PROMs)**

The distribution of patient-reported disability severity, depressive symptoms, and erectile dysfunction (ED) are shown in Supplementary Figures 2–4. According to the Oswestry Disability Index (ODI), 82.2% of patients had severe-to-exaggerated functional disability, with 39.4% severe, 35.2% very severe (crippled), and 7.6% bed-bound or exaggerated symptoms (Supplementary Fig. 2). Depression assessment (PHQ-9) revealed that nearly half (42.2%) reported moderate-to-severe symptoms, with 24.5% moderate, 15.2% moderately severe, and 2.5% severe (Supplementary Fig. 3). Among male patients, ED assessed via the IIEF-5 questionnaire was prevalent, affecting 76.6%, with 35.1% classified as severe, 18.9% moderate, and 12.6% mild-to-moderate (Supplementary Fig. 4).


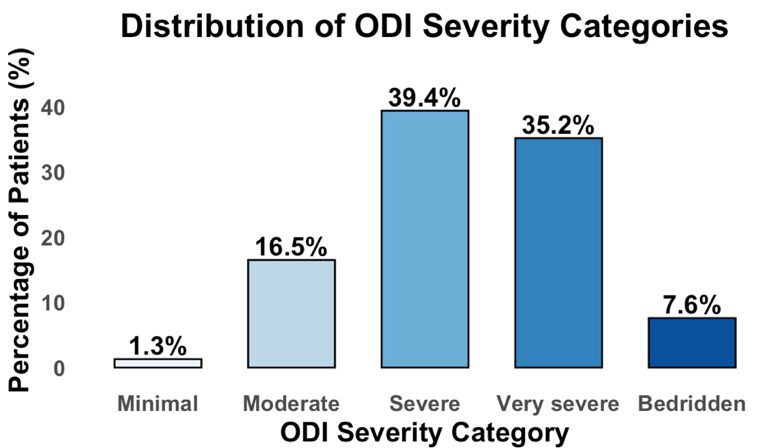


**Figure 2: Distribution of ODI severity categories among patients (N=247).**

Bar graph illustrating the percentages of patients in each Oswestry Disability Index (ODI) severity category: minimal (0–20%), moderate (21–40%), severe (41–60%), very severe (61–80%), and bedridden (>80%).


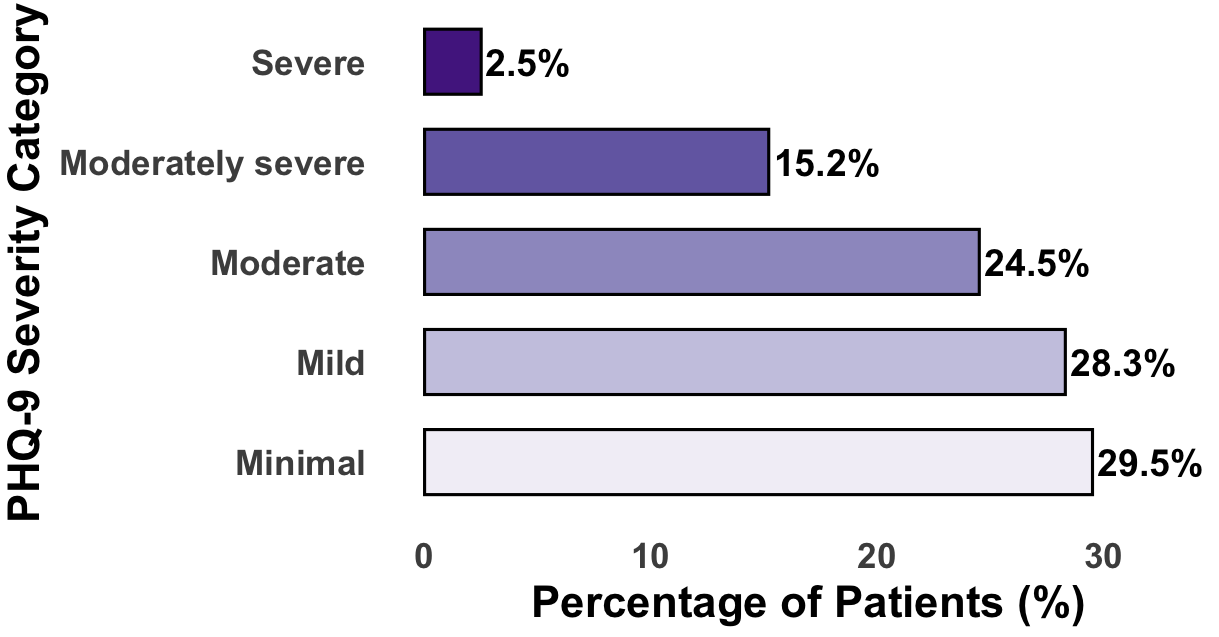


**Figure 3: Distribution of depression severity (PHQ-9) among patients with lumbar pain (N=237).**
Horizontal bar graph demonstrating the percentage of patients in each depression severity category measured by the Patient Health Questionnaire-9 (PHQ-9).


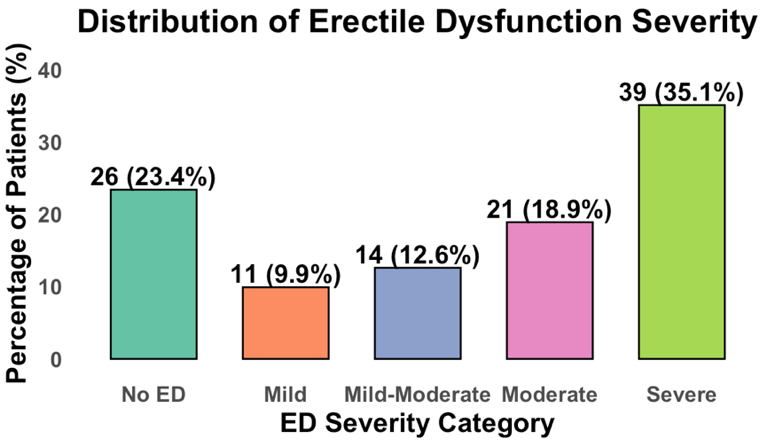


**Figure 4: Distribution of erectile dysfunction (ED) severity among male patients with lumbar pain (N=111).**

Vertical bar graph showing percentages and absolute numbers of male patients across ED severity categories according to the International Index of Erectile Function (IIEF-5).
